# Harnessing 3D models to uncover the mechanisms driving infectious and inflammatory disease in the intestine

**DOI:** 10.1186/s12915-024-02092-9

**Published:** 2024-12-31

**Authors:** Diana Micati, Sara Hlavca, Wing Hei Chan, Helen E. Abud

**Affiliations:** 1 Department of Anatomy and Developmental Biology, Monash University, Clayton, VIC 3800 Australia; 2 Development and Stem Cells Program, Monash Biomedicine Discovery Institute, Monash University, Clayton, VIC 3800 Australia

**Keywords:** Intestinal diseases, Inflammatory bowel disease, Modelling, Organoid co-culture, Epithelium-mesenchyme interaction, Epithelial-microbe interaction, High-throughput screening, Personalised medicine

## Abstract

Representative models of intestinal diseases are transforming our knowledge of the molecular mechanisms of disease, facilitating effective drug screening and avenues for personalised medicine. Despite the emergence of 3D in vitro intestinal organoid culture systems that replicate the genetic and functional characteristics of the epithelial tissue of origin, there are still challenges in reproducing the human physiological tissue environment in a format that enables functional readouts. Here, we describe the latest platforms engineered to investigate environmental tissue impacts, host-microbe interactions and enable drug discovery. This highlights the potential to revolutionise knowledge on the impact of intestinal infection and inflammation and enable personalised disease modelling and clinical translation.

## Background

### Authentic models are required to decipher the complexity of intestinal disease

**Fig. 1:**
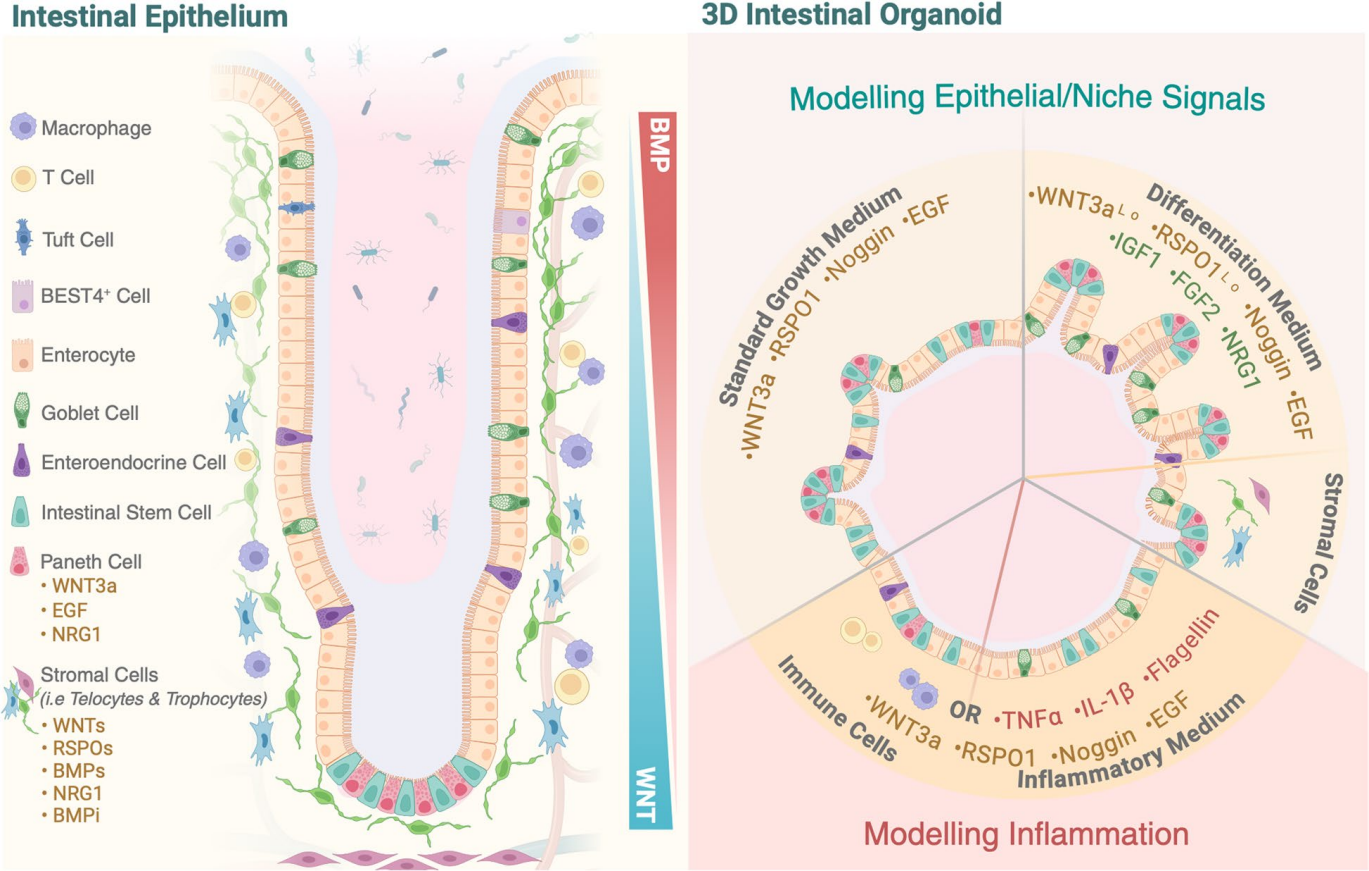
Modelling epithelial and niche signals in intestinal organoids to mimic cellular diversity. The intestinal epithelium consists of stem and differentiated cells and is surrounded by mesenchymal stromal and immune cells that secrete key signals to support epithelial self-renewal, proliferation and differentiation. This complexity can be modelled in organoids by adding specific niche factors or cell types. Briefly, organoids are typically cultured in standard growth medium. To drive cell differentiation and diversity, organoids are cultured in a differentiation medium supplemented with reduced WNT3a (WNT3a^Lo^) and RSPO1 (RSPO1^Lo^), and with niche factors like NRG1, or with stromal cells only. To model inflammation, organoids can be exposed to an inflammatory medium where standard growth medium is supplemented with different pro-inflammatory cytokines and bacterial components, or by introducing immune cells while maintaining standard organoid culture conditions

The intestinal tract undergoes significant morphological changes from development through to adulthood [1–3] generating regions with distinct cellular composition and function in digestion and nutrient absorption within the small intestine and colon [4]. The intestine is lined with a rapidly regenerating epithelial layer where resident stem cells located within crypts contribute to tissue homeostasis [5] and repair following injury [6, 7] (Fig. 1). The apical surface of the epithelium is continuously exposed to microbes and by-products in the lumen that can cause tissue damage contributing to diseases such as inflammatory bowel diseases (IBD). IBD and other diseases can vary between individuals [8, 9], suggesting disease modelling should be conducted using pre-clinical systems that can test responses from multiple patients. Immortalised cell lines [10] and animal models have been extensively utilised and have contributed to our understanding of cellular mechanisms and signalling pathways but they fail to recapitulate numerous physiological and phenotypic aspects of human disease [11].

The ability to grow primary human intestinal cells as three-dimensional (3D) organoids [12, 13] has opened new avenues to more accurately model numerous aspects of organ physiology including cellular fate transitions, cell-to-cell communication and host-microbe interactions. Adult stem cell (ASC)-derived organoids were initially established from the mouse small intestine [13] and consist solely of epithelial cells. These organoids have the remarkable capacity to self-organise to closely resemble crypts in vivo where stems cells are intercalated between Paneth cells in buds and regions of proliferation and differentiation can be clearly identified [13, 14] (Fig. 1). Epithelial organoids also retain the regional-specific characteristics from where they were isolated enabling the mechanisms underlying regional identity to be examined [15]. Although intestinal organoids better resemble endogenous tissue compared to two-dimensional (2D) models, human intestinal organoid models still do not fully recapitulate the cellular diversity and differentiation states found in vivo [12, 16, 17]. It is also clear that mechanical signals and spatial interactions with other cell types including stromal, immune, endothelial and lymphatic cells modulate epithelial cell fate [7, 18–30]. In this review, we discuss the latest approaches in utilising organoids to characterise the impact of cellular signals and microbes on epithelial tissue, engineering approaches to advance organ-on-a-chip models, innovations in high-throughput screening, organoid phenotyping and artificial intelligence (AI). We further discuss these current advances in the field, evaluating challenges and applications to clinical translation.

## Main text

### *Recapitulating the cellular diversity of the intestinal epithelium *in vitro

The adult small intestinal epithelium is composed primarily of absorptive enterocytes with secretory cells including goblet, Paneth, enteroendocrine and tuft cells [31, 32] (Fig. 1). Crypts that invaginate into the underlying mesenchyme and house stem and progenitor cells are present throughout the GI tract, while villi that protrude into the lumen contain differentiated cells are only present in the small intestine [31]. In contrast, the adult colonic epithelium is predominantly composed of enterocytes and goblet cells, with a notably higher proportion of goblet cells compared to the small intestine [33, 34], reflecting its role in mucin secretion and water absorption. Additionally, the colon lacks Paneth cells but retains other secretory cell types such as enteroendocrine cells, which contribute to local hormonal signalling [33, 34]. The absence of villi in the colon results in a different structural organisation of the epithelium, which consists of deeper crypts and a thicker mucosal layer [35]. Overall, while both the small intestine and colon serve crucial roles in digestion and absorption, their cellular compositions and structures reflect their distinct physiological functions.

Recent single-cell and spatial transcriptomic analyses have revealed cellular composition and transcriptional profiles vary considerably along the proximal–distal axis in humans. This includes the characterisation of cell types such as Bestrophin 4 (BEST4) cells, a subset of the absorptive lineage that is present in humans, not mice [4, 36–39] (Fig. 1). Stem cells fuel the renewal of the intestinal lining every 4–5 days [40], with new cells proliferating within the transit-amplifying (TA) zone before terminally differentiating and migrating upwards along the crypt-villus axis [5] (Fig. 1).

A key breakthrough in 2009 demonstrated that intestinal organoids could be established from crypts or isolated Lgr5^+^ stem cells from mice, and when embedded in gel containing extracellular matrix, self-organised into stem and differentiation zones [13]. This study recapitulated supportive niche signals by the addition of R-spondin1, Noggin and WNT3a that are normally secreted from surrounding cell types [18, 23, 25, 30] (Fig. 1). These organoids supported the growth of cells that maintained apical-basal polarity, modelled the cellular heterogeneity of in vivo tissue and retained the transcriptional profile typical of their regional origin [15]. The methodology was then adapted to culture human tissue [12]. Although tissue can be grown, there are many more difficulties and variabilities in growing human intestinal organoid cultures, with cellular composition, level of maturity and organisation not fully representative of the repertoire of cell types and spatial organisation in the adult human intestine [12, 16, 41, 42]. Improvements in culture conditions are developing [17], but require further optimisation to generate a highly reproducible culture system that faithfully mirrors that of in vivo tissue. For instance, a recent study demonstrated that incorporating insulin-like growth factor 1 (IGF1) and fibroblast growth factor 2 (FGF2) into the culture medium promoted simultaneous multi-lineage differentiation and self-renewal of human intestinal organoids [43] (Fig. 1). These and other environmental factors discussed below can influence organoid phenotypes (Fig. 1). These changes can be quantitatively assessed through different screening protocols with machine learning aiding both tracking of organoids and image analysis [44–47].

### Modelling interactions in the tissue microenvironment

In recent years, efforts to understand the regulation of intestinal stem cell plasticity, critical for epithelial barrier repair, mesenchymal remodelling and regeneration following damage, have driven continuous advancements in intestinal organoid co-culture with various resident cell populations and supportive scaffolds. Traditionally, elucidating aspects of cell–cell interactions relied on animal models; however, they fail to identify short-distance interactions. In vitro co-culture systems bridge this gap, providing a clearer understanding of key contributors by simplifying the complex in vivo environment. Several studies have demonstrated the feasibility of integrating induced pluripotent stem cell (iPSC)-derived organoids with neurons [48, 49], vascular endothelial cells [48, 50] and adipocytes [51]. However, limited studies are available on the co-culture of patient-derived organoids (PDOs) with niche cells, primarily due to the technical complexities involved in such cultures (reviewed in [52]). Some of these challenges include determining the type of interaction between the desired cell types (also defined as direct or indirect co-cultures), selecting the most suitable co-culture medium to support the growth of all relevant cell types and choosing the correct plasticware. For instance, transwells are mainly used for studying cell–cell interactions [53], while ultra-low attachment plates are better suited to grow apical-out organoids, useful for investigating microbe-epithelial cell interactions [54]. In contrast, tissue culture-treated plates are used to grow and expand organoids within an extracellular matrix, closely mimicking the tissue environment [13]. Building on this, selecting the correct matrix is crucial. Utilising synthetic matrices in place of the commonly used, but poorly defined basement membrane extracts like Matrigel, offers the potential to more accurately replicate the native tissue environment [55, 56]. This can be achieved by fine-tuning the biophysical properties of the synthetic matrix, such as stiffness, which is frequently heterogeneous in diseased tissues (reviewed in [57, 58]).

Despite these limitations, several significant studies have identified key regulatory signals that impact the activity of epithelial cells by co-culture with immune cells, fibroblasts and microbial components. A detailed overview of co-culturing epithelial cells with various components of the microenvironment is presented in the sections below.

### Intestinal organoid co-cultures with stromal cells

Intestinal stem and progenitor cells are tightly regulated by niche factors produced by stromal cells that reside underneath the epithelium (reviewed in [18]). Fibroblasts are essential drivers of epithelial regeneration following damage [6, 7, 23–25]. Therefore, defining the activity of signalling molecules that are crucial for mucosal repair may have therapeutic potential in treating degenerative intestinal diseases, such as IBD, where the epithelial barrier is impaired.

Stromal cells have been identified as potential sources of key signals by molecular profiling that has identified the presence of WNT and BMP agonists and antagonists, in stromal cells along the crypt-villus axis [23–25, 30, 59, 60] (Fig. 1). Other signals such as members of the epidermal growth factor (EGF) family of ligands, secreted from stromal cells, have also been directly applied to organoid cultures in order to model in vivo biological activity [7, 17, 42, 61] (Fig. 1). Sources of these essential signals in the mesenchyme were proposed to be supplied by stromal cells marked by expression of platelet-derived growth factor receptor alpha (PDGFRɑ), subepithelial telocytes, that localise in close proximity to cells within crypts and villi, and trophocytes located near the base of crypts marked by expression of CD81 (Fig. 1). The function of these cells has been verified by co-culture experiments, enabling the assessment of stromal-epithelial interactions. Most methods involve embedding 3D organoids in Matrigel on top of a layer of fibroblasts, allowing direct contact for morphological assessment [62, 63]. While this method facilitates direct interaction, it poses challenges in separating the compartments for downstream analysis, such as secretome profiling. To address this, transwell systems can be used, where organoids are seeded in Matrigel in the apical compartment, and stromal cells as monolayers in the basal compartment [64]. Building on this, co-culturing intestinal organoids with specific stromal cell types has proven successful in enabling the long-term culture of organoids in the absence of growth factors that are typically essential for their survival (Fig. 1). For example, telocytes [23, 25, 60, 65] and PDGFRɑ-low trophocytes, source of crucial stem cell growth factors GREM1, WNT2b and R-spondin3 [30], fully support organoid growth in culture ex vivo. These studies have identified the function of specific cell types and insight into the contributions of specific growth factors. However, this approach fails to recreate the complexity of the stromal niche that establishes a spatial morphogen gradient in vivo [30] (Fig. 1). This has been partially addressed with intestinal assembloid cultures that combine diverse stromal cell types with epithelial organoids to produce self-organising structures with distinct stem and differentiated cellular compartments [66]. This highlights the importance of bi-directional signals between epithelial and stromal cells.

### Intestinal organoid co-cultures with immune cells

In addition to stromal cells, immune cells play a crucial role in maintaining homeostasis by protecting against pathogens, regulating inflammation and supporting epithelial barrier function. As key components of the intestinal mesenchyme, immune cells are activated in response to injury and infection, playing a crucial role in protecting and repairing the epithelium. Understanding the interaction between immune and epithelial cells is critical for identifying novel therapeutic targets, particularly in the context of anti-inflammatory therapies. Disruption in this communication can have significant pathophysiological consequences, emphasising the importance of studying these interactions in disease.

A common approach to modelling inflammation as seen in diseases like IBD involves supplementing the organoid medium with one or a cocktail of pro-inflammatory molecules. Several studies on mouse and human intestinal organoids identified that adding tumour necrosis factor alpha (TNFα), interleukin-1 beta (IL-1β), lipopolysaccharide (LPS), Flagellin and interferon-gamma (INF-γ), either individually or in combination, induces an inflammatory response that mimics certain aspects of IBD [67–70] (Fig. 1). While this approach allows for direct study of specific cytokines’ effects on organoids, it lacks the complexity provided by co-culturing methods.

Establishing immune cell-human organoid co-cultures is challenging; however, several methods have been developed using various immune cells such as T-lymphocytes, innate lymphoid cells, macrophages, neutrophils, dendritic cells and intraepithelial lymphocytes [26, 71–74]. Depending on the aim of the experiment, co-cultures can be allogeneic or autologous, where donors of immune and epithelial cells are different or the same, respectively. For translational purposes, researchers rely on autologous immune cells, obtained from different sources. Peripheral blood mononuclear cells (PBMCs) are commonly employed due to their accessibility. Tumour immunology can also be modelled with co-culture of immune cells and organoids derived from cancer patients [22, 75]. While immune cells, such as T-lymphocytes, can be isolated from biopsy samples [76], this method presents challenges, necessitating collection of multiple samples from the same donor to ensure enough material is generated and demanding more specialised skills. Co-culture protocols involve the pairing of immune cells with either 3D organoids [21], which preserve the key features of the original tissue, or alternatively, with organoid-derived monolayers (Fig. 1) [74]. The choice of co-culturing method depends on the experimental goals. For instance, co-culturing immune cells with 3D organoids is ideal when the focus is on understanding how immune cells influence the spatial organisation and behaviour of epithelial cells in a more in vivo-like environment. For example, the integration of T-cells into the epithelial cell layer was observed in 3D organoids alongside changes in T-cell morphology and epithelial gene expression [77]. Alternatively, co-culturing immune and epithelial cells as monolayers using a transwell system separates luminal and basal compartments, while establishing the natural polarity of epithelium. This configuration is particularly useful for studying concurrently host–pathogen interactions, immune responses to enteric pathogens and immune-epithelial communication, as it enables the examination of both apical and basolateral signalling. For example, human macrophages seeded on the basal side of organoid-derived epithelial monolayers have been observed to generate projections that stretch through the epithelial layer when bacteria were seeded on the apical surface [78]. The ability to segregate these compartments enhances the ability to measure secreted factors and observe interactions on the basal and apical sides of the epithelium that represent in vivo tissue dynamics.

When considering immune-epithelial cell co-culture, all cell types require specific culture medium. This makes it often difficult to find a balanced combination of factors that allows survival of all populations within the same experiment. For instance, co-culturing T-cells with epithelial cells is feasible but challenging. Activation of T-cells requires supplementing the co-culture medium with interlukin-2 (IL-2) [21], which, while necessary for T-cell function, it promotes epithelial cell maturation and differentiation [79]. This shift in cell composition in organoids can complicate studies targeting stem cell function. Another significant challenge is the use of foetal bovine serum (FBS). To minimise the impact of unknown FBS components on epithelial cells, organoids should ideally be cultured in media with low FBS (< 1%) or in FBS-free conditions [80]. However, immune cells typically require FBS to prevent stress responses like autophagy, which can impair their cytokine production [81]. Despite these challenges, ongoing advancements in co-culture techniques, such as the development of more refined media formulations, and the potential for machine learning to guide selection of growth conditions [82] offer promising solutions. These innovations may allow for precise control over the cellular microenvironment, reducing unwanted influences on both epithelial and immune cell behaviour.

### Utilising organoids to assess epithelial-microbe interactions

**Fig. 2:**
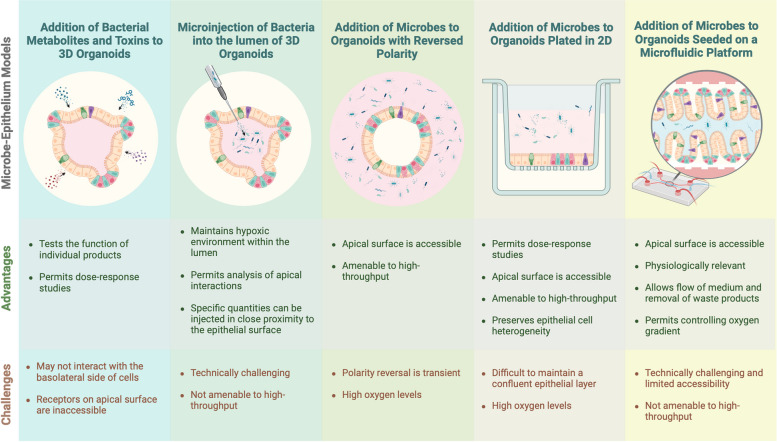
Current models of microbe-epithelial interactions. Bacteria and toxins can be introduced to organoids using different approaches depending on the type of interaction being investigated. This includes the use of 3D models, microinjection, apical-out organoids, organoids grown as a 2D monolayer and microfluidic platforms

In addition to the internal intestinal microenvironment, defined by the intricate interactions amongst immune, stromal and epithelial cells, understanding how the external microenvironment influences epithelial cells is essential for promoting tissue regeneration and maintaining homeostasis. Growing evidence suggests that elements of the human microbiome play a significant role in either supporting a healthy epithelial barrier or exacerbating intestinal diseases including IBD [83]. Most of this evidence has been correlative, but with recent breakthroughs in the capability to culture a diversity of microbes from the human intestinal tract [84], functional studies are now possible. To gain mechanistic insight into how enteric microbiota, both commensal and pathogenic, interact with epithelial cells, protocols have been developed to culture human intestinal organoids with microbes [85]. For instance, Lamers et al. highlighted the potential of using human intestinal organoids as an infection model for studying the SARS-CoV-2 virus [86]. To accurately replicate the interactions and infection pathways between epithelial cells and microbes, selecting the appropriate co-culture model is essential. Organoids typically self-organise such that the apical surface is located on the inside representing the lumen. This requires microinjection of microbes into the lumen or strategies to “flip” cell polarity [87, 88], so the apical surface is on the outside to accurately mimic epithelial-microbe interactions. There are also co-culture protocols that utilise organoids plated in 2D in transwells to facilitate analysis of host-microbe interactions. These approaches are summarised in Fig. 2 and are discussed in more detail below.

(i) Studying metabolites and toxins.

Microbes that reside within the GI tract can modulate epithelial tissue directly or through the secretion of metabolites and toxins. The complexity of these interactions can be difficult to decipher but the specific impact of individual factors can be assessed by direct addition to organoids. Metabolites such as short chain fatty acids and bile acids can modulate the proliferation of intestinal stem cells [89], and the activity and receptor specificity of bacterial toxins can be experimentally tested in organoids [90].

(i) Microinjection.

Microbes may be injected directly into the lumen if maintenance of the structural integrity of the intestinal organoid is required. This is typically performed under a microscope via a specialised needle that can puncture individual organoids and dispense drugs or bacteria [85]. To facilitate the ease of injection and visualisation of the dispense of bacteria, the organoids are usually first induced into a cystic shape by promoting stem cell proliferation. Cystic organoids have a relatively larger lumen, a thinner epithelial layer and less luminal debris, all of which are crucial to enable injection efficiency. Similar to other co-culture models, dedicated media conditions must be established beforehand to fulfil the needs of both microbial and epithelial cell growth. Optimising the multiplicity of infection is also essential to prevent bacterial overgrowth within the lumen [85]. Furthermore, antibiotics may be added to the medium to restrict bacterial growth external to the organoids [91]. This methodology has been utilised by several groups to study infection of *Listeria monocytogenes* in mouse small intestinal organoids [92]; *Helicobacter pylori* in mouse and human gastric organoids [93, 94]; *Salmonella enterica* serovar Typhimurium (*S.* Typhimurium) in mouse small intestinal organoids [95] and human iPSC-derived intestinal organoids [96]; and *Cryptosporidium parvum* oocytes in ASC-derived intestinal organoids [97], amongst others. One group successfully transplanted two different bacteria (*Escherichia coli* and *Yersinia pseudotuberculosis*, each fluorescently labelled red and green, respectively) into a single organoid and visualised both within the same lumen [91]. This was subsequently repeated with whole faecal-derived microbiota, highlighting the scalability of this method depending on the desired output [91]. In addition to infection, microinjection has been used to measure barrier permeability by quantifying the amount of fluorescently labelled dextran that is transferred from the lumen of the organoid in a given amount of time [98]. In contrast to other culturing methods such as 2D organoid culture in which bacteria are added to the medium, microinjecting directly into the lumen permits researchers to control the amount of bacteria within each organoid. Another advantage of microinjection as a methodology to study epithelial-microbe interactions is the hypoxic environment within an organoid lumen which supports the growth of anaerobic bacteria and is thus more biologically relevant [91]. Despite these benefits, organoid microinjection is technically challenging, requires advanced equipment and is not amendable to high-throughput experimentation as only one organoid can be microinjected at one time [54].

(i) Apical-out organoid.

In addition to microinjection, several groups have recently reversed the epithelial polarity within organoid models so that the apical surface is facing outwards and is in contact with the medium [54, 99, 100]. As epithelial polarity is largely regulated via extracellular membrane (ECM) proteins within the basement membrane in vivo or Matrigel in culture, intestinal organoid polarity is rapidly reversed to an apical-out state if transferred to a suspension medium lacking any ECM components. Supplementation of basement membrane extract to these apical-out organoids in suspension restored the basal-out phenotype in a dose-dependent manner [54, 99]. Importantly, Co et al. determined that these apical-out organoids retained key features of basal-out organoids including intact barrier integrity, cellular differentiation and selective diffusion [54, 99]. Furthermore, when co-cultured with the pathogenic bacteria *S.* Typhimurium, the bacterium preferentially bound to the apical surface (now accessible) and compromised barrier function of the organoids [54, 99]. These results highlight the potential of apical-out organoids in studying the relationship between the intestinal epithelium and pathogenic bacteria that act through the apical surface. Although these apical-out organoids maintain a 3D structure and do not require the microinjection of microbes into the lumen, they are hindered by their short life span in the reversed state, the propensity to aggregate and the inability to change the medium while in suspension [101].

(iv) 2D monolayer organoids.

Although a key benefit of organoid models is the ability to exist in three dimensions and thus recapitulate a physiologically relevant environment, for certain research questions, a 2D model may be more appropriate. This can be achieved by seeding intestinal crypts on a thin layer of dilute Matrigel [87, 88], or more commonly, directly on transwell cell culture inserts [102–106]. The resulting monolayer maintains key features of 3D organoid models including epithelial polarisation [88, 102, 103], proliferation and self-renewal [88, 105], cellular heterogeneity [88, 102–106], barrier integrity [88, 102, 103, 105, 106] and a pathophysiological response to various stimuli [102, 103, 106]. For instance, Ettayebi et al. reported the first in vitro culture of Norovirus in human epithelial cells using 2D monolayer organoids [107], which could not be achieved in transformed cell lines [108]. Roodsant et al. infected human monolayer organoids with a pathogenic virus and bacterium, Enterovirus A71 (EV-A71) and *Listeria monocytogenes*, respectively, to demonstrate their effectiveness in studying epithelial-microbe interactions [102]. Both virus and bacterium demonstrated the ability to translocate from the apical to the basal side of the epithelial monolayer, with EV-A71 disrupting barrier function and *L. monocytogenes* inducing inflammation in the process [102]. This was similarly observed by Holthaus et al. who co-infected mouse monolayer organoids with two parasitic protozoans, *Toxoplasma gondii* and *Giardia duodenalis*, with a marked loss of barrier integrity observed only in the presence of *G. duodenalis* [103]. This highlights the capacity of 2D organoid cultures to easily facilitate infection with a variety of microbes and organisms, and thus reinforces this method as a possible alternative to 3D organoid models to study disease with a strong microbial component such as IBD. However, despite their advantages, 2D organoids still present challenges for long-term culture. Prolonged culture results in low oxygen tension, which leads to cellular stress and altered secretory cell differentiation [101, 109]. To address this, an air–liquid interface (ALI) method using a transwell system can alleviate oxygen stress by exposing only the basolateral side of the cells to the culture medium while keeping the apical side exposed to air [109, 110]. This approach promotes a higher degree of differentiation and produces long-lived models ideal for studying chronic infections [111–113]. For example, Boccellato et al. successfully used an ALI co-culture system to study *H. pylori* infection for up to 4 weeks [113]. Another challenge in co-culturing systems is the selection of a suitable growth medium, as different cell types and microbes often require specialised conditions for growth. However, transwell systems, which separate the apical and basal compartments, allow for the optimisation of culture conditions for both bacteria and epithelial cells, creating a microenvironment that better mimics in vivo conditions [114]. With recent advances in microfluidic technology, more physiologically relevant conditions, such as oxygen gradient, pH level and specific growth factors, can be better recapitulated.

### Advances in bioengineering organoids

Despite these advances in culture conditions to improve the physiological relevance of organoid models, they still lack some fundamental features of the in vivo environment due to their growth as spherical structures. This includes the tissue geometry and physical forces, fluid flow across the luminal surface and variations in oxygen concentration. These forces are important for the patterning of tissue and measuring bacterial interactions [20, 115]. To address this, intestine-on-a-chip model systems have been generated in which intestinal epithelial cells (IECs) or whole organoids are seeded on a microfluidic platform to which endothelial cells, immune cells and the patient’s microbiome may be added [116, 117] (Fig. 2). Typically, the microfluidic platform is composed of two channels between which the IECs are seeded in a polarised manner so that one channel is representative of the lumen and the other of the basolateral surface [118]. Therefore, unlike in organoid models, the apical side is easily accessible in intestine-on-a-chip model systems for administration of bacteria, cytokines and drugs in a physiologically relevant manner [119]. Peristaltic-like movement can be artificially induced in intestine-on-a-chip models via the addition of a vacuum, while the flow of medium over the cells more accurately reflects the in vivo intestinal environment by providing similar mechanical cues and allows for removal of waste products produced by the IECs [116, 117]. Furthermore, by controlling the oxygen content of the supplied medium, an artificial oxygen gradient may also be established to more accurately reflect the in vivo intestine in which varying oxygen gradients are essential for nutrient absorption and maintenance of a complex microbiota [117, 118].

Due to their complexity, intestine-on-a-chip models are limited by their physical and financial demands and are thus only suitable for more nuanced questions that require the presence of the complete microenvironment, mechanical stress from peristalsis or a controllable oxygen gradient while still being accessible in vitro. These advantages of intestine-on-a-chip models make them highly valuable for researching complex, multifactorial diseases like IBD, but perhaps are limiting when more direct interactions or high-throughput screening approaches are being investigated. Intestine-on-a-chip models are already being utilised to answer a broad range of questions relating to intestinal biology and disease. For example, Liu et al. established a controllable oxygen gradient across a microfluidic platform to successfully co-culture the highly oxygen sensitive anaerobic bacterium *Bifidobacterium bifidum* with aerobic human intestinal epithelial cells (Caco-2) [120]. Beaurivage and colleagues were also successful in generating a microfluidic intestine-on-a-chip culture from Caco-2 cells which were likewise stimulated with inflammation-inducing cytokines to model IBD [121]. Within 4 days of establishment, the Caco-2 cells had already formed an effective barrier that was subsequently lost after treatment with inflammatory cytokines [121]. In the following year, Beaurivage and colleagues further optimised this methodology by using PDOs to more accurately model the heterogenous intestinal epithelium and introduced monocyte-derived macrophages which were induced to produce cytokines via addition of LPS and IFN-γ [119]. Induction of an inflamed state was subsequently assessed via RNA-sequencing which confirmed upregulation of genes relating to the innate immune response in microfluidic cultures treated with LPS and IFN-γ [119]. Kim et al. cultured pathogenic and non-pathogenic *E. coli* on an intestine-on-a-chip system [122]. From this, Kim et al. observed that non-pathogenic *E. coli* had no detectable effect on the intestinal epithelium, while pathogenic *E. coli* induced loss of barrier integrity and tissue architecture in the intestine-on-a-chip models, a result not detected with LPS endotoxin alone [122]. This paper highlights the importance of external factors (i.e. the gut microbiota and the immune system) when modelling infection or features of IBD, and thus intestine-on-a-chip systems provide an alternative approach to studying intestinal disease and facilitating and drug discovery when compared to strictly epithelial systems such as organoid models.

Furthermore, these intestine-on-a-chip systems may be optimised by combining the technology with 3D bioprinting [123]. 3D bioprinting involves the deposition of a 3D scaffold (using biomaterial inks) onto which heterogenous cell cultures are seeded [124–126] or alternatively, cells may be directly 3D printed (using bioinks) to generate complex microenvironments in vitro [125]. Recent work on 3D printed intestinal tissue arranged in either a polarised monolayer with protrusions [127–129] or in a cylindrical tube [130, 131] has highlighted the effectiveness of establishing and maintaining a selectively permeable barrier with this methodology [101, 127–129, 132], the potential for co-culture with non-epithelial cells or bacteria/viruses [128, 129, 131] and the possibility to replicate a diseased state [132, 133]. Analysis of data in these systems also benefits from utilising AI.

Ultimately, the goal of bioprinting is to generate whole organs in vitro for research or transplantation purposes; however, this is currently limited by its resolution capacity to generate small structures such as capillaries [126]. Until this is overcome, bioprinting can be used instead to help generate intestine-on-a-chip systems and enhance reproducibility. Thus, these next generation engineered models incorporate spatial and mechanical cues to establish human systems that mimic human mucosa even more closely, and make the complex architecture of the in vivo intestine more accessible in vitro for the study of multi-cellular diseases such as IBD [134]. This includes reproducing crypt structure, morphogen gradients, luminal flow and cell turnover. These bioengineered systems can model tumour initiation and disease states [135].

### Utilising high-throughput organoid screening to facilitate personalised medicine

**Fig. 3:**
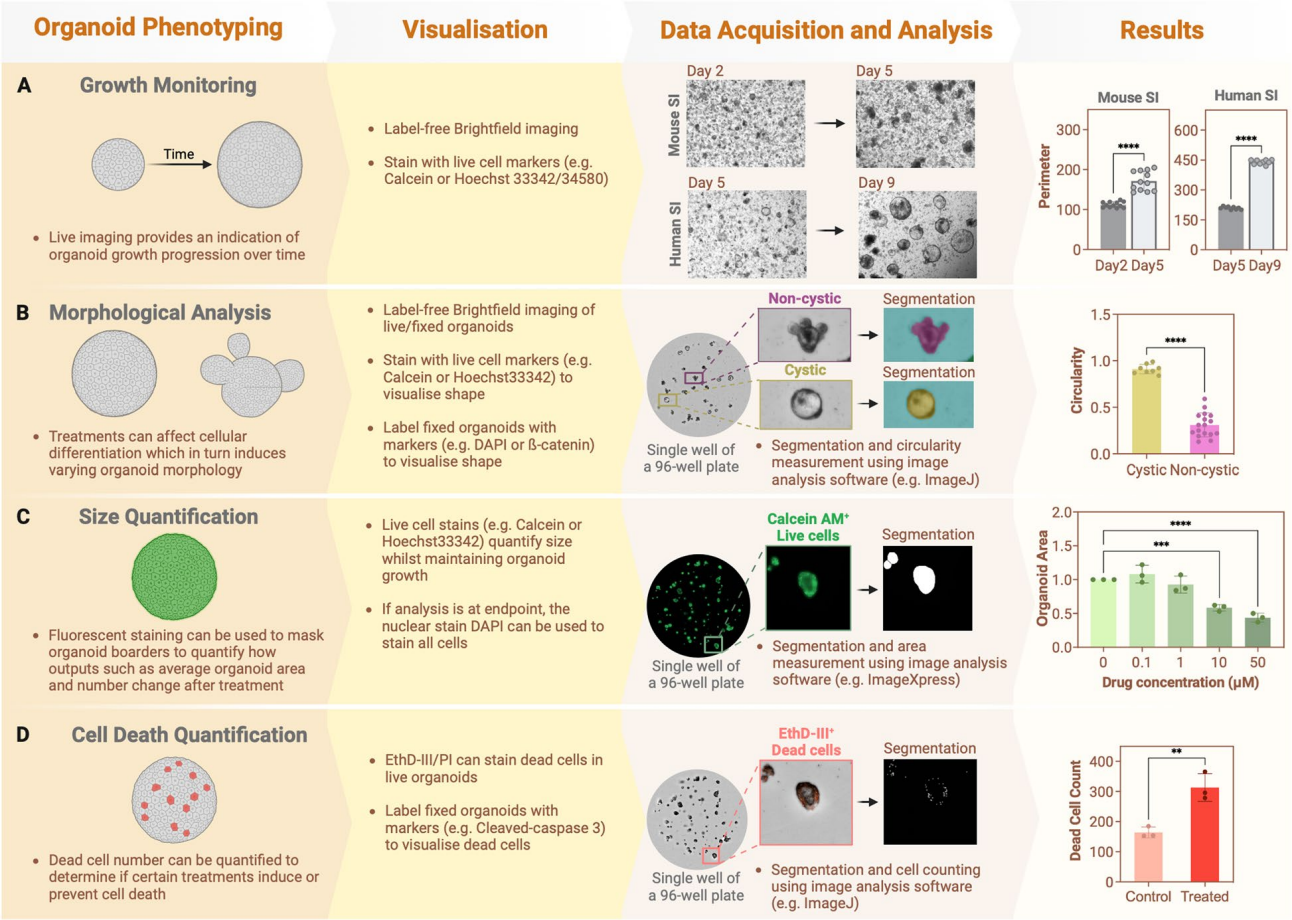
Current screening methods for quantifying organoid growth and morphology. Phenotypic changes in organoids over time or in response to specific treatments can be quantified by assessing growth, morphology, size and viability. These changes can be visualised using brightfield imaging of live/fixed organoids or by labelling live/fixed organoids with different cell markers or stains, depending on the experimental objective. High-content imaging systems (e.g. ImageXpress Pico Automated Cell Imaging System) enable data acquisition, with analysis performed using software such as ImageJ. **A** Growth, measured by perimeter, can be plotted to show changes over time. In this example, growth of mouse small intestinal (SI) organoids was measured at day 2 compared to day 5, and human SI organoids at day 5 compared to day 9 (paired *t*-test). **B** Morphology can be assessed through circularity which determines the degree of differentiation. In this example, morphological heterogeneity of mouse SI organoids is observed within the same well. Organoids exhibiting a cystic morphology are less differentiated (value closer to 1), whereas non-cystic organoids are more differentiated (value closer to 0) (paired *t*-test). **C** Organoid size can be measured using brightfield imaging or by labelling organoids with a fluorescent marker for better segmentation analysis to enable the evaluation of size changes in response to specific treatments. In this example, individual human colorectal cancer organoids have been masked based on their Calcein AM signal and the resulting area quantified (expressed as fold change from the untreated control, one-way ANOVA). **D** Viability analysis is performed to assess the proportion of live vs dead cells in organoids under different treatment conditions. Cell death can be measured by using different cell death stains such as ethidium homodimer III (EthD-III). In this example, the number of dead cells in colorectal cancer organoids upon addition of chemotherapy drug has been plotted to evaluate treatment impact on survival (unpaired *t*-test)

A valuable feature of PDOs is the ability to perform chemical screening to identify potential therapeutics across a broad range of patients. To this extent, human organoid models have an advantage over cell lines and animal models as they more readily recapitulate human genetic diversity. One of the first drug screens performed on an organoid biobank was performed by van de Wetering and colleagues using patient-derived colorectal tumour (*n* = 22) and normal organoids (*n* = 19) [136] where they successfully tested 83 compounds with varying degrees of sensitivity utilising the CellTitre-Glo (CTG) cell viability assay. More recently, Luo et al. identified four potential hits out of a total of 139 compounds which reduced the viability of high-risk colorectal adenoma organoids [137]. Phenotyping approaches to analyse impacts of drugs is summarised in Fig. 3. Alternatively, with more specific questions, a single compound may be tested on a larger cohort of organoids to determine the effect of a single drug of interest across a range of genetic backgrounds. For example, Nishimura and colleagues have utilised human normal colon organoids (supplemented with cytokines and bacteria to replicate IBD) to investigate the effect of the novel drug, KAG-308, in relation to IBD [138]. Using this model system, the authors found that KAG-308 supressed many of the inflammation-associated genes and instead supported the intestinal stem cell population as observed by the increase in proliferation and organoid establishment after treatment with KAG-308.

**Fig. 4:**
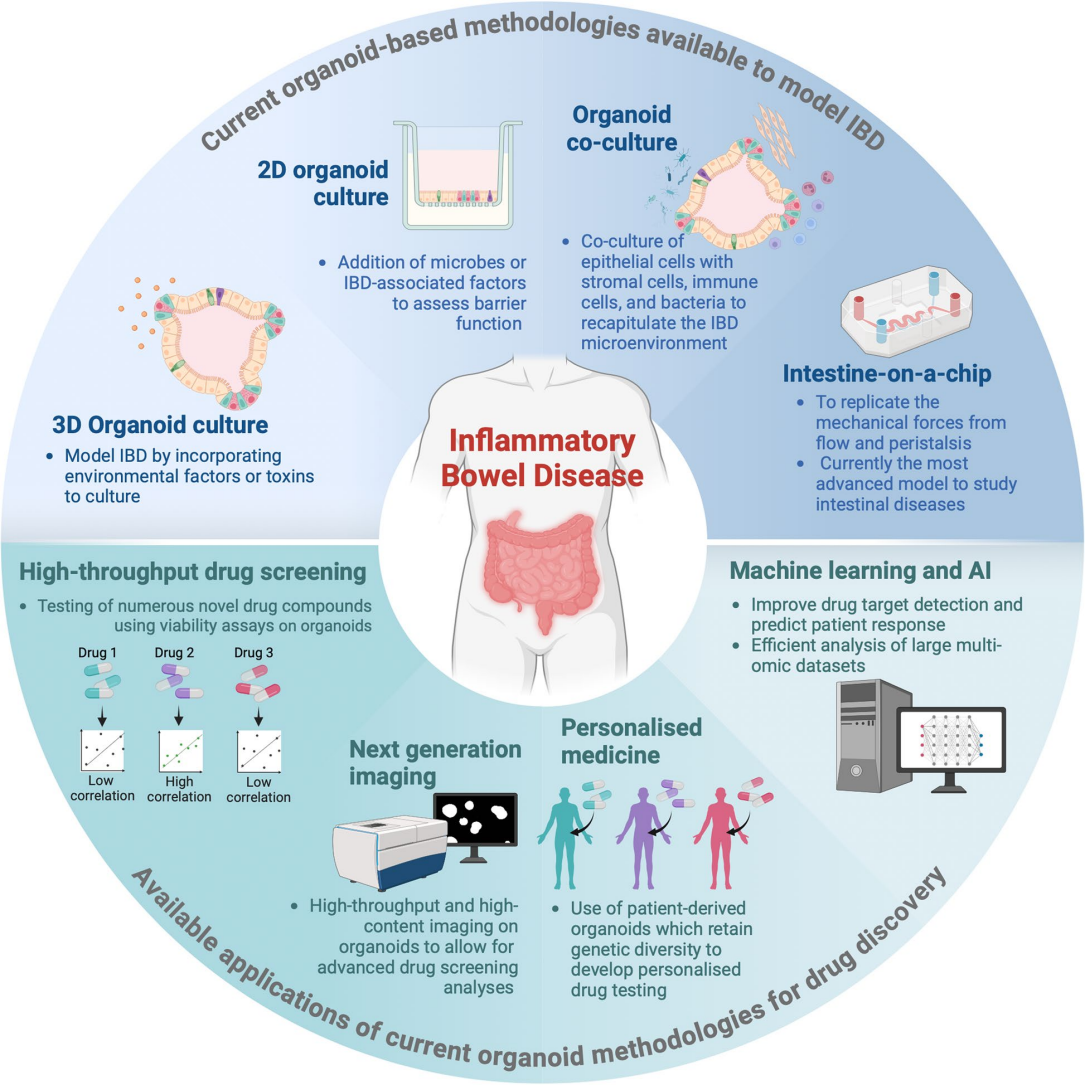
Overview of advancing methodologies to model IBD. Different approaches to model IBD include traditional or co-culture organoid systems, 2D organoid culture and intestine-on-a-chip. Currently, these models serve as a tool for more accurate personalised medicine, next generation imaging and high-throughput drug screening, while advancements in AI and machine learning could further enhance the quantity and quality of data generated from these models

Metabolism-based assays such as CTG are common outputs of organoid drug screens; however, drug response in organoid models can additionally be measured through image-based phenotypic screens which quantify the effect of a drug based on a visual change in the organoids (Fig. 3) [139, 140]. Typically, image-based phenotypic screens use fluorescent markers to measure organoid size as a representation of cell viability which has been confirmed to be comparable to a CTG assay [140]. However, alternative fluorescent markers may be chosen depending on the desired read-out of the assay, e.g. live/dead cells, proliferative cells, cell type-specific markers or a combination of several markers for multiplex imaging, and thus image-based phenotypic screens can provide a more complex picture of the effect of candidate drugs on organoid models [139] (Fig. 3). Furthermore, if combined with the emerging advances in machine learning that can both integrate multi-omic data and predict drug response, this process could become even more informative and streamlined in the future [141, 142] (Fig. 4).

## Conclusions

### Future perspectives

Since the initial discovery of conditions that support the growth and self-organisation of native intestinal tissue, there have been significant advances in both the range and complexity of systems to analyse the function of the human gut. As highlighted in Fig. 4, these innovative models have enormous potential for translational applications including the discovery of new drug and bacterial treatments for IBD and screening for effective treatments for pathological infections such as *Clostridium difficile*. The ability to establish organoids efficiently from individual patients, advances in high-throughput screening, AI and machine learning paves the way for personalised medicine approaches to improve effective patient treatment.

## Data Availability

No datasets were generated or analysed during the current study.

## Abbreviations

2D: Two dimensional
3D: Three dimensional
AI: Artificial intelligence
ALI: Air-liquid interface
ASC: Adult stem cell
BEST4: Bestrophin 4
CTG: CellTitre-Glo
ECM: Extracellular matrix
EGF: Epidermal growth factor
EthD-III: Ethidium homodimer III
EV-A71: Enterovirus A71
FBS: Foetal bovine serum
FGF2: Fibroblast growth factor 2
IBD: Inflammatory bowel disease
IEC: Intestinal epithelial cell
IGF1: Insulin-like growth factor 1
IL-1β: Interleukin-1 beta
IL-2: Interleukin-2
INF-γ: Interferon-gamma
iPSC: Induced pluripotent stem cell
LPS: Lipopolysaccharide
PBMC: Peripheral blood mononuclear cell
PDGFRα: Platelet-derived growth factor receptor alpha
PDO: Patient-derived organoid
*S*. Typhimurium: *Salmonella enterica* Serovar Typhimurium
SI: Small intestine
TA: Transit-amplifying
TNFα: Tumour necrosis factor alpha
